# The complete mitochondrial genome of a bagrid catfish, *Tachysurus nudiceps*, and its phylogenetic implications for the classification of the bagrid genera

**DOI:** 10.1080/23802359.2022.2057253

**Published:** 2022-04-01

**Authors:** Syuri Hashimoto, Ryosuke Kakehashi, Tsuyoshi Mori, Chiaki Kambayashi, Shigefumi Kanao, Atsushi Kurabayashi

**Affiliations:** a Faculty of Bio-Science, Nagahama Institute of Bio-Science and Technology, Shiga, Japan; b Lake Biwa Museum, Kusatsu, Japan

**Keywords:** Mitogenome, next-generation sequencing, *Tachysurus nudiceps*, Bagridae

## Abstract

The complete sequence of the mitochondrial genome (mitogenome) of *Tachysurus nudiceps* (family Bagridae; order Siluriformes) was determined using next-generation sequencing. The composition of its mitogenome is the same as that observed in most other vertebrates and consists of 37 genes, an L-strand replication origin and a control region. As in previous studies, our phylogenetic analyses revealed that many of the bagrid genera are not monophyletic, emphasizing the necessity for reviewing and revising the taxonomy of this family at the genus level.

*Tachysurus nudiceps* (Sauvage, 1883) of the family Bagridae is a freshwater catfish endemic to Japan. This catfish is listed as a ‘vulnerable’ or ‘near threatened’ species by local governments with jurisdiction within its natural distribution range, specifically in western Japan (Association of Wildlife Research and Envision 2007; Takano et al. 2016). Thus, detailed distribution surveys of this species are desirable, and the targeted eDNA method is known to be effective for such a distribution survey (Bylemans et al. 2019). However, there is little genetic information for this species, and no species-specific primers have been developed for eDNA surveys. In this study, we determined the entire mitochondrial DNA (mtDNA) sequence of this species, with the main aim of designing specific primers in the future.

The *T. nudiceps* specimen was collected from Kamo River, Kyoto, Japan (35.0305 N, 135.7709 E) and deposited at the Lake Biwa Museum [https://www.biwahaku.jp/english/ contact: Shigefumi Kanao, kanao-shigefumi@biwahaku.jp] under the voucher number LBM-1210059075. The *T. nudiceps* total DNA was extracted from the liver tissue using a ‘Mitochondrial DNA isolation kit’ (BioVision, CA, USA) and sequenced on the DNBSEQ-G400 platform using a 200-bp paired-end procedure. From the resultant raw data, low-quality nucleotide sites (< Q30) were deleted using the ‘sickle’ software (Joshi and Fass 2011), and the whole mtDNA was assembled from the remaining data using NOVO Plasty 4.3 (Dierckxsens et al. 2017) with the *Pelteobagrus tokiensis* mtDNA (AB054127) used as the reference. Gene annotation was performed using the MITOS WebServer (Bernt et al. 2013) and inaccurate gene boundaries were corrected by visual observation.

The *T. nudiceps* mtDNA sequenced (DDBJ/EMBL/GenBank accession number: LC664019) is 16,529 bp in length and contains the typical mitogenome components of vertebrates, that is, 37 genes (13 protein-coding genes [PCGs] and two ribosomal and 22 transfer RNA genes) and two prominent non-coding regions, namely light-strand replication origin (O_L_) and control region (CR). The arrangement of these components is the same as that observed typically in vertebrates (Boore 1999). The nucleotide composition is 31.1, 25.9, 15.2, and 27.8% for A, T, G, and C, respectively. Similar to some other animal mitogenomes, in the *T. nudiceps* mtDNA, a non-canonical GTG start codon (Desjardins and Morais 1991) was observed in the CO1 gene. In addition, incomplete stop codons, T and TA, which can form a complete TAA stop codon by post-transcriptional polyadenylation (Anderson et al. 1981), are found in six PCGs (CO2-3, Cytb, and ND2-4).

We performed phylogenetic analyses using 13 PCGs (11,376 bp) of the 41 bagrid species for whom the complete mitogenome data were available. In the analyses, we applied a partitioning strategy (Nylander et al. 2004) for the 13 PCG dataset. The best-fit partitioning scheme and optimal substitution models for the selected partitions (see legend of Figure 1) were estimated by ModelFinder (Kalyaanamoorthy et al. 2017) implemented in IQ-tree version 1.6.12 (Nguyen et al. 2015) with Bayesian information criterion (Schwarz 1978). Maximum-likelihood (ML) and Bayesian inference (BI) tree reconstructions were performed using IQ-tree and MrBayes v3.2.7 (Ronquist et al. 2012), respectively. The ML and BI analyses results showed the same tree topology (Figure 1), and this topology was very similar to that reported by Liu et al. (2019). It is known that the genus-level taxonomy of the family Bagridae is controversial (Liu et al. 2019). Following this, only two genera in our tree, *Horabagrus* and *Mystus,* formed their own clade, and the monophyly of each genus *Hemibagrus*, *Pelteobagrus*, *Pseudobagrus*, and *Tachysurus*, was not supported. For example, the sister species of *T. nudiceps* is *Pel. tokiensis* (ML Bootstrap [MLBP] and Bayesian Post Probability [BPP] = 92 and 1.0, respectively) rather than the other *Tachysurus* species. Our results emphasize the necessity of revising the genus-level classification of this catfish family. Furthermore, several authors have proposed the species belonging to the genera *Pelteobagrus*, *Pseudobagrus*, and *Tachysurus* into one genus (Ng and Freyhof 2007; Liu et al. 2019). Our results support this proposition. However, prior to making the taxonomic modifications, further phylogenetic analyses based on the mitogenomic data of more bagrid taxa, especially of the type species of each problematic genus, should be carried out.

**Figure 1.**
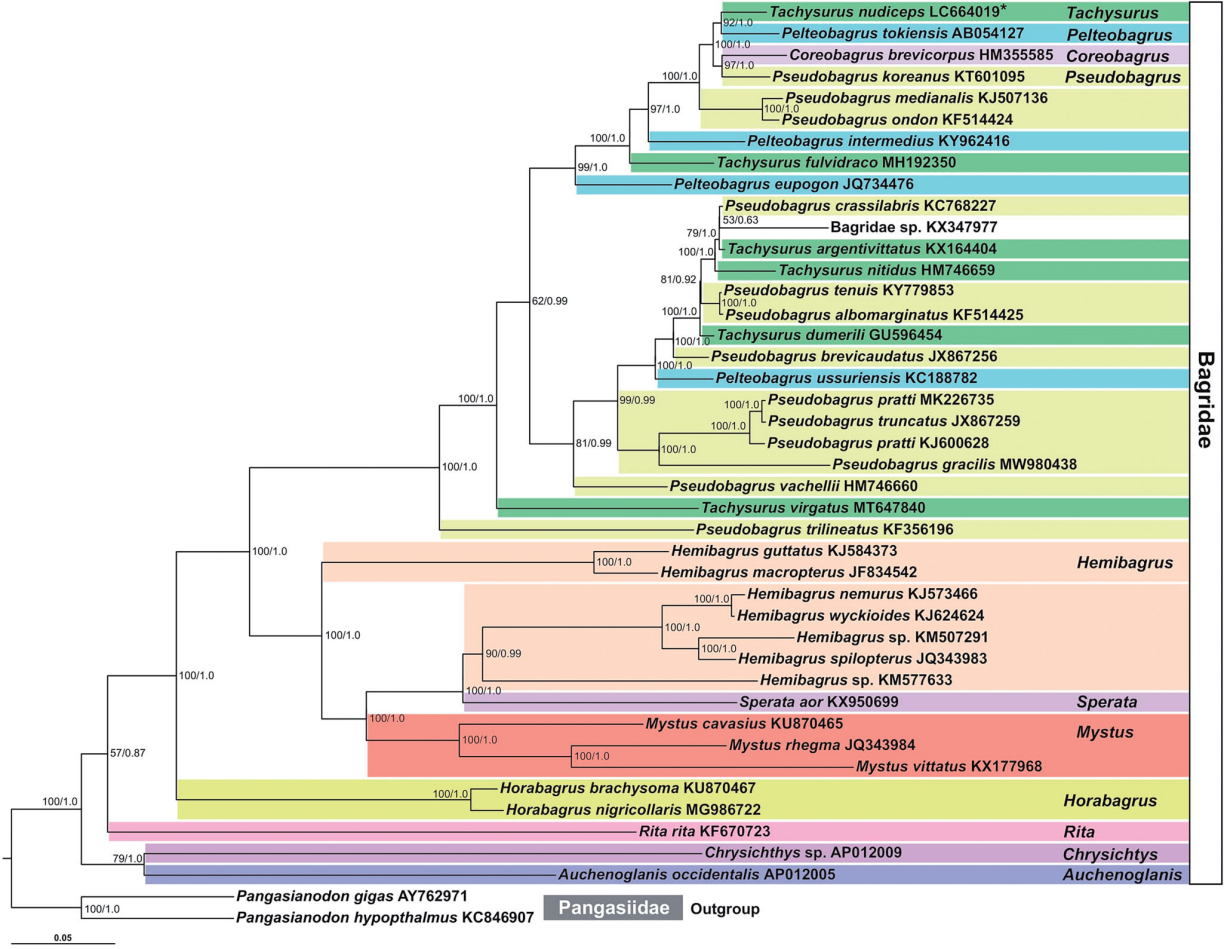
Phylogenetic tree of bagrid catfishes based on mitochondrial protein-coding gene sequences. The ML tree of 41 bagrids reconstructed from the 13 PCG sequences with the best-fit partitioning scheme is shown. *indicates *Tachysurus nudiceps* of which mtDNA was sequenced in this study. The partitioning scheme and optimal substitution models for the selected partitions were estimated by IQ-tree (partition 1 = ATP6 + Cytb + ND1-5 with substitution model GTR + F+G + I, partition 2 = ATP8 + CO1-3 with GTR + F+G + I, and partition 3 = ND6 with HKY + F+I + G4). The numbers at the nodes indicate nonparametric ML Bootstrap values (left) calibrated by 1,000 pseudo replicates and Bayesian Post Probabilities (right) calibrated by four independent MCMC runs for 10 million generations (sampling frequency is one per 1,000 generations) without first one million samples (i.e. 10% burn-in). Two *Pangasianodon* species (family Pangasiidae) were used as the outgroup. The scientific names were followed to FishBase (Froese and Pauly 2021).

## Author contributions statement

A.K. and S.H. conceived the study. T.M. and S.K. collected the specimen. S.H., R.K., T.M., and C.K. carried out the experiments and/or data analyses. A.K., C.K., and S.K. wrote the manuscript with contributions from all authors. All authors approved the final manuscript and agreed to be accountable for all aspects of the study.

## Ethical approval

The ethics committee of Nagahama Institute of Bio-Science and Technology (NIBST) approved this research (No.102).

## Data Availability

The genome sequence data that support the findings of this study are openly available in GenBank of NCBI at [https://www.ncbi.nlm.nih.gov/nuccore/LC664019] under the accession no LC664019. The associated BioProject, SRA, and Bio-Sample numbers are PRJDB12761, DRA013229 and SAMD00433587, respectively.
